# The Rocky Road from Experience to Expression of Emotions—Women’s Anger About Sexism

**DOI:** 10.1007/s42761-021-00081-7

**Published:** 2021-11-24

**Authors:** Julia Sasse, Jolien A. van Breen, Russell Spears, Ernestine H. Gordijn

**Affiliations:** 1 Faculty of Behavioral and Social Sciences, University of Groningen, Groningen, The Netherlands; 2 Max Planck Institute for Research On Collective Goods, Kurt-Schumacher-Str. 10, 53113 Bonn, Germany; 3 Institute of Security and Global Affairs, Leiden University, Leiden, The Netherlands

**Keywords:** Anger, Emotion expression, Sexism, Identification, Feminism

## Abstract

We investigated women’s anger expression in response to sexism. In three studies *(N*s = 103, 317, and 241), we tested the predictions that women *express* less anger about sexism than they *experience*—the anger gap—and that the anger expressed by women is associated with instrumental concerns, specifically perceived costs and benefits of confronting sexism. To estimate the specificity of the proposed gap, we compared women’s anger reactions to men’s anger reactions as well as anger reactions to sadness reactions. Across studies, we found support for the anger gap, that is, lower anger expression than experience, and the gap was more pronounced for women than for men (Study 3). Surprisingly, a gap also occurred in sadness reactions. Regarding instrumental concerns, there was converging evidence that expressed anger was negatively associated with individual costs. We also investigated whether anger expression can be encouraged through women’s identification with feminists (Studies 1 and 2) and support by other women (Study 2); yet, we found no evidence. We conclude that, to understand women’s—and men’s—reactions to sexism, it is critical not to mistake their emotion expression for how they really feel, but instead to also consider strategic concerns.

**Supplementary Information:**

The online version contains supplementary material available at 10.1007/s42761-021-00081-7.

Exposure to sexism gives rise to a range of emotions, but people might not always be able to express those freely, for various reasons. Anger, in particular, can be both effective *and* restricted: It may provide an emotional base to mobilize action (van Zomeren et al., 2012), and it can signal objection and stimulate conciliatory tendencies from those discriminating (de Vos et al., 2013). At the same time, when women express anger, they often experience a backlash (Rudman & Glick, 1999), as they violate gender prescriptions (Fischer & Evers, 2010). If women make a trade-off between potential benefits and costs, they may refrain from (fully) expressing the anger they experience in response to sexism—resulting in an *anger gap*. To date, we know little about how these concerns come together. In this paper, we investigated the *anger gap* and scrutinized instrumental concerns in anger expression in response to sexism.

## Instrumental Concerns of Anger Expression

The perception that one’s in-group is treated unjustly often triggers the *experience of anger* (Ellsworth & Scherer, 2003; Mackie et al., 2000). Critically, anger experience does not necessarily translate into *anger expression*, as they constitute distinct emotion dimensions that can be regulated independently (Greenaway & Kalokerinos, 2019). We argue that expression may be regulated with *instrumental concerns* in mind (see Tamir, 2009, 2016), and we considered two concerns particularly relevant for women’s anger expression in response to sexism.

The first of these instrumental concerns is the *collective benefits* the group may obtain from expressions of anger. Based on the communicative function of emotions (Fischer & Manstead, 2008; van Kleef, 2009), anger expression towards in-group members may aid a shared understanding of perceived injustice, thereby stimulating collective action. Expressions of anger towards an antagonist out-group can communicate that a valued relationship has been damaged and even trigger conciliatory tendencies in the out-group (de Vos et al., 2013).^1^ Consequently, expressing anger in response to sexism should help to reduce gender discrimination, which benefits the in-group. We therefore expected that concerns about collective benefits should positively predict women’s anger expression in response to sexism.

However, women may also anticipate *individual costs* by expressing anger. Women’s anger contravenes social role beliefs, prescribing women to be kind and caring (Eagly & Wood, 2016; Evers et al., 2011; Fischer & Evers, 2010; Plant et al., 2000). Further, women who behave in role-incongruent ways face backlash (see Hercus, 1999). The prospect of violating gender prescriptions or backlash may discourage women from expressing anger. For these reasons, we expected that the more women are concerned with such individual costs, the less anger they express in response to sexism.

## Specificity and Magnitude of the Gap

For a comprehensive understanding of the hypothesized anger gap, it is critical to establish its specificity, with regard to group (women) and emotion (anger) and to consider potential variations in its magnitude.

To estimate emotion-specificity of the gap, we contrasted anger with sadness. Like anger, sadness arises in response to injustice (Mikula et al., 1998), but being sad is more in line with women’s gender role prescriptions (Brody & Hall, 2010). Thus, because it is less likely to trigger backlash, sadness should be less costly to express. Consequently, for women, we expected the experience and expression of sadness to be more in line than of anger, leading to a smaller gap.

Moreover, we expected that the gap is specific to *women*’s anger reactions to sexism. For men, the context of sexism is less impactful, and therefore we might expect them to respond less strongly than women. Also, being angry is in line with gender role prescriptions for men (Brody & Hall, 2010), and therefore less costly to express than for women. Thus, we predicted that men show a smaller anger gap than women.

The magnitude of the anger gap might also vary between women. Generally, individuals highly identified with their group experience group-based emotions more strongly than those less identified (Gordijn et al., 2006; Smith et al., 2007). In the context of gender and sexism, in particular the feminist identity should be relevant as it is concerned with the *social position* of women (van Breen et al., 2017). Consequently, we expected feminist identification to affect experienced anger in response to sexism. More importantly, given that expressed anger may serve as a signal against sexism, we also predicted that higher feminist identification leads to more anger expression. Thus, for high feminist identifiers, the anger gap should be smaller, with both anger experience and expression being relatively high compared to non-feminists.

Finally, we investigated whether the anger gap can be attenuated through *support* from other women. Arguably, learning that other women confront sexist behavior could create a sense of support among likeminded women. As this has been identified as a main driver of collective action (Spears et al., 2002; van Zomeren et al., 2004), we expected similar motivating effects for anger expression. Consequently, we predicted that support by other women motivates anger expression, reducing the gap.

## The Present Research

In three studies, we confronted female participants with a (fictitious) sexist blog post and assessed their anger reactions to it. We predicted anger expression to be lower than experience (the anger gap) and to reflect instrumental concerns of individual costs and collective benefits. Additionally, we predicted more anger expression, and hence a smaller gap, the more women identify with feminists (Studies 1 and 2) and in case of support (Study 2). As a benchmark for the anger gap, we explored sadness reactions in Studies 1 and 2 and preregistered a larger gap for anger than sadness in Study 3. There, we also compared women’s reactions to men’s reactions and predicted a larger anger gap and stronger concerns about individual costs for women.

All materials and data, including measures not reported here, can be found here https://osf.io/fvrz4.

## Study 1

### Methods

#### Participants

We recruited 103 female participants through an online paid participant pool run by the University of Groningen (*M*_age_ = 21.85, *SD*_age_ = 2.74). Participants were compensated with two euros. The majority of participants studied (97; 3 worked, 3 missing values) and was Dutch (44; overall, participants came from 24 different countries).

We computed sensitivity analyses to determine the size of effects we were able to detect in our sample. Setting statistical power to .90 and assuming a correlation of *r* = .50 between measures of experienced and expressed anger, in repeated-measures analyses of variance (ANOVAs), we were able to detect effects of *f* = .14 and *f* = .19 (when considering feminist identification and women identification as a control). In a multiple linear regression with three predictors, we were able to detect an effect of *f*^2^ = .14.

#### Procedure and Design

The study was approved by the ethics committee of the University of Groningen and conducted in accordance with ethical guidelines. We obtained consent from all participants, and they were fully debriefed at the end of the study. Participation was anonymous and could be terminated at any point in time.

First, we measured participants’ identification with feminists. Next, we presented them with the sexist blog post. After administering checks to ensure that participants perceived the blog post as sexist, we measured anger reactions to it. In our analysis, we used experienced and expressed anger as two levels on a repeated measure, which we call *Anger Gap* henceforth.

#### Exposure to Sexism

We asked participants to read a blog post, allegedly written by a man as a reaction to the International Equal Pay Day. In this post, the author strongly advocates for traditional gender roles with respect to work and family. He justifies the existence of the gender pay gap with men and women having different preferences and skills and arguing that “ladies are good at being nurses and kindergarten teachers so you should not strive for becoming engineers.” The blog post concluded by reducing the woman’s role to staying at home and keeping her husband happy. We further included two supportive statements by other men below the post to ensure that this opinion was perceived as being held not only by the author as a single person but as shared by other men.

#### Measures

If not specified otherwise, participants gave their responses on 7-point scales.

#### Identification

We measured identification with feminists with four items (e.g., “I identify with feminists,” Cronbach’s *α* = .96). For exploratory purposes, we used the same four items to measure identification with women and replaced the word “feminist” with “women” (Cronbach’s *α* = .78; procedure adapted from van Breen et al., 2017).

#### Checks

After participants had read the sexist blog post, we measured the extent to which they perceived the post as sexist, as promoting gender equality, and agreed or disagreed with the author (scales from − 50, *fully disagree* to 50, *fully agree,* with 0 being neutral).

#### Anger About the Sexist Opinion

Participants rated the extent to which they *experienced anger* about the blog post with a single-item measure (“I experience anger”). On a separate page, we measured *anger expression intentions* by asking participants to what extent they would express anger if they were to respond to the blog post.

Note that the measures for experienced anger and anger expression intentions were presented together with four other emotions (sadness, amusement, humiliation, fury) to disguise that we were particularly interested in anger.

We used responses to the sadness items to explore the emotion-specificity of the proposed gap.

#### Individual Costs and Collective Benefits

We assessed the extent to which different concerns influenced emotion expression with 15 items. Most importantly, some were intended to capture individual costs and collective benefits. We computed a factor analysis (generalized least squares extraction, oblique rotation), which revealed three factors: The first factor represented *individual costs* (six items, e.g., “I want to avoid being seen as a troublemaker”; eigenvalue 3.66, explaining 24.44% of variance). The second factor represented efforts for *collective benefits* (three items, e.g., “I want to speak up against gender discrimination”; eigenvalue 2.29, explaining 15.29% of variance), while the third factor was rather concerned with *intergroup harmony* (three items, e.g., “I want to bring men and women closer together”; eigenvalue 1.02, explaining 6.79% of variance). We decided to exclude three items from further analyses: We excluded two items (“I want to avoid appearing weak” and “I want to avoid being seen as a traditional woman”) because they loaded on both the first and the second factor. The item “Voicing my opinion would make me feel better” loaded on the collective benefits factor, but as it is rather concerned with an individual motive, we decided to exclude it.

With respect to our hypotheses, individual costs (Cronbach’s *α* = .78) and collective benefits (Cronbach’s *α* = .74) are most relevant. Descriptive statistics and correlations with anger for the intergroup harmony scale and excluded items are reported in the supplementary material.

### Results

#### Identification

Participants were moderately identified with feminists (*M* = 3.70, *SD* = 1.67) and quite highly identified with women (*M* = 5.72, *SD* = .99). The two types of identification were weakly correlated, *r* = .28, *p* = .004.

#### Checks

As intended, participants perceived the blog post as sexist, *M* = 32.13, *SD* = 28.88; significantly different from the scale midpoint of 0, on a scale from − 50 to 50; *t*(102) = 12.13, *p* < .001, *d* = 1.11 and harming efforts towards achieving gender equality, *M* =  − 40.82, *SD* = 19.29; *t*(102) =  − 21.47, *p* < .001, *d* = 2.12. Importantly, participants disagreed with the author’s opinion, *M* =  − 35.90, *SD* = 24.01; *t*(102) =  − 15.18, *p* < .001, *d* = 1.50.

#### Anger About the Sexist Opinion

Our first goal was to establish the anger gap. Participants reported being very angry about the blog post (*M* = 5.29, *SD* = 1.68), but, as expected, they were not prepared to express anger to the same extent (*M* = 4.96, *SD* = 1.79). A repeated-measures ANOVA revealed that this discrepancy was statistically significant, *F*(1,101) = 5.61, *p* = .02, *η*_p_^2^ = .05, supporting the idea of the anger gap.

We then investigated systematic differences in the anger gap due to feminist identification. We re-ran the repeated-measures ANOVA and included feminist identification as a continuous predictor (mean centered). We also included women’s identification as a control, which allowed us to estimate the unique effect of feminist identification. The anger gap remained significant, *F*(1,97) = 7.63, *p* = .01, *η*_p_^2^ = .07, and was not qualified by feminist identification, *F*(1,97) = 0.10, *p* = .75, *η*_p_^2^ = .001. Thus, different from our hypothesis, feminist identification did not attenuate the anger gap. Feminist identification did however have a main effect on anger (both experienced and expressed), such that higher identification led, on average, to more anger, *F*(1,97) = 13.18, *p* < .001, *η*_p_^2^ = .12.

#### Costs and Benefits Concerns

What instrumental concerns impacted anger expression? In line with our hypotheses, bivariate correlations showed that participants intended to express less anger the more they took individual costs into account and more when they cared about collective benefits (Table 1). To assess unique contributions of each concern, we conducted a multiple linear regression with experienced anger, individual costs, and collective benefits as predictors and anger expression intentions as outcome. Individual costs retained a unique negative effect on anger expression intentions above and beyond experience, *B* =  − 0.33, *SE* = 0.10, 95% CI [− 0.52, − 0.14], *t*(96) =  − 3.38, *p* = .001, anger experience, *B* = 0.62, *SE* = 0.09, 95% CI [0.44, 0.79], *t*(96) = 6.99, *p* < .001; collective benefits *B* = 0.24, *SE* = 0.13, 95% CI [− 0.02, 0.49], *t*(96) = 1.85, *p* = .07.

**Table 1:** Descriptive statistics and correlations for experienced and expressed anger and sadness, individual costs, and collective benefits in Study 5

							Correlations		
		*N*	*M*	*SD*	1	2	3	4	5
1	Experienced Anger	103	5.29	1.68	-				
2	Experienced Sadness	103	4.12	1.94	.45**				
3	Anger Expression Intentions	102	4.96	1.79	.69**	.35**			
4	Sadness Expression Intentions	102	3.36	1.88	.22*	.51**	.24*		
5	Individual Costs	100	3.80	1.29	− .09	− .06	− .29*	.60	
6	Collective Benefits	100	5.28	1.15	.55**	.32**	.49**	.12	− .06

^**^p < .001; *p < .01

#### Is the Gap Emotion-specific?

To shed light on the question whether the gap is specific to anger, we contrasted anger reactions with sadness reactions. We ran a repeated-measures ANOVA with the factors Gap (experience vs. expression) and Emotion (anger vs. sadness). The main effects of gap, *F*(1,101) = 20.01,* p* < .001, *η*_p_^2^ = 0.17, and emotion, *F*(1,101) = 60.32, *p* < .001, *η*_p_^2^ = 0.37 were significant. The interaction between both factors was significant as well, *F*(1,101) = 4.60, *p* = .03, *η*_p_^2^ = .04. Interestingly, simple main effects showed that the gap was more pronounced for sadness, *F*(1,101) = 17.54,* p* < .001, than for anger, *F*(1,101) = 5.61, *p* = .02 (see Table 1 for descriptive statistics).

### Discussion

Study 1 provided evidence for the proposed anger gap in women’s responses to sexism. The gap was not attenuated by feminist identification, though high feminist identifiers experienced and intended to express more anger than low identifiers. Critically, anger expression intentions were associated positively with collective benefits and negatively with individual costs, with the latter retaining a unique effect when considered simultaneously, reflecting the conflicting instrumental concerns associated with anger expressions. Interestingly, there was also a gap in sadness reactions to sexism that was, contrary to our reasoning, even more pronounced than the anger gap.

## Study 2

With Study 2, we sought to replicate and extend the findings of Study 1. Instead of exclusively relying on anger expression intentions, we obtained written responses to the sexist comment that were rated for anger expression. Moreover, in addition to feminist identification, we investigated whether support through critical reactions of other women to the sexist behavior can motivate anger expression.

### Methods

#### Participants

We recruited 331 female participants through an online paid participant pool run by the university. Out of those, 317 completed 75% or more of the study and were hence included in the sample (*M*_age_ = 23.13, *SD*_age_ = 5.21). Participants were compensated with two euros. The majority of participants was Dutch (114; overall, participants came from 46 different countries).

The size of our sample enabled us to detect relatively small effects, setting *α* = .05 statistical power of .90 and, where required, the correlation between experienced and expressed anger to *r* = .67 (based on results in Study 5). To test the anger gap and the effects of in-group criticism, feminist identification, as well as women’s identification in a mixed ANOVA, we were able to detect effects of *f* = .10. For multiple linear regressions with three predictors, the smallest detectable effect size was *f*^2^ = .05.

#### Procedure and Design

Procedure and materials were largely identical with those in Study 5. Again, participants first reported their identification with feminists and women and then read the sexist blog post. Immediately afterwards, we applied the checks to ensure that the post was perceived in the intended manner and assessed experienced anger responses to it. On the next page, we then introduced in-group support as a between-subjects factor and randomly assigned participants to one of two conditions (support no vs. yes). In both conditions, participants saw two comments in support of the blog post, allegedly written by two male readers, as in Study 5. In the support condition, participants then read two additional comments, by two female readers (e.g., “Jeff, guess what, we are not here to ‘serve’ you!!! We deserve the same opportunities as men…”).^2^ In the no-support condition, participants did not read these comments criticizing the author.

This was followed by manipulation checks, measures of expressed anger, individual costs, and collective benefits, and an open text box for written replies to the post. These written responses were later rated by independent trained raters to obtain a second expressed anger measure.

#### Measures

 Measures were largely identical to Study 1 with some adjustments and additions.

#### Identification

We measured feminist and women identification with the same items as in Study 5 (*α* = .86/ = .95).

#### Checks

We asked participants to rate the extent to which the comments supported or opposed the opinion presented in the blog post and their own opinion. For both items, we used a slider scale ranging from − 50 (labeled *oppose*) to + 50 (labeled *support*).

#### Anger About the Sexist Opinion

We measured experienced anger with two items (“I am angry/ irritated about the blog post”; *r* = .67, *p* < .001). We used the same adjectives to measure *anger expression intentions* (“I would express that I am angry/ irritated about the blog post”; *r* = .67, *p* < .001).

In order to go beyond intentions, we obtained a second measure of *verbally expressed anger* from written responses. Here, participants were asked to formulate their own comment freely in reply to the blog post. Participants were instructed to reply how they would reply in real-life online interactions. To ensure that participants had complied with the task, two female independent raters, blind to conditions, judged whether participants had provided an actual reply, a hypothetical reply, or no reply; in case of disagreement a third female rater was consulted. The large majority of participants provided actual replies (269), few formulated hypothetical replies (14), and 34 participants did not write a reply at all.^3^ Replies differed in length from single words to 296 words, with an average length of 66.70 words (*SD* = 54.84).

Most importantly, the two raters were trained to rate anger expression in these comments. Specifically, they were asked to judge “Overall, how much anger is expressed in the reply?” on a scale from 1 (*not angry at all*) to 4 (*very angry*). Replies that were rated 4 contained strong language or anger-related words (e.g., “… I have seldom felt such frustration and anger about something written on the internet. Jeff, this is clearly sexist, humiliating bullshit”). Replies rated 3 conveyed angry sentiment through explicit criticism of the author without the use of anger-related words (e.g., “I bet all the women here are smarter and better suited than you to become anything they want. You are just a sad man trying to maintain gender stereotypes to make yourself feel better!”). Replies rated 2 expressed moderate levels of anger through disapproval of the post (e.g., “… Maybe it’s not all women’s dream pursuit, but if some choose and have the ability to go down that path, what makes you think you should say something about? Mind your own business. …”). Replies rated 1, instead, were phrased neutrally with regard to anger (“I think every person can choose for him or herself what he or she wants. This is not all about gender but of the person you wanna be.”). The intraclass correlation coefficient indicated that absolute agreement between both raters was moderate, *r* = .57, 95% CI [0.45, 0.67].

#### Sadness about the Sexist Opinion

In order to test whether the gap is specific to anger, we used sadness as a comparison emotion. Along with the anger items, participants responded to two items assessing experienced sadness (“I am sad about/down because of the blog post”, *r* = 51, *p* < .001) and two items assessing sadness that they intended to express (“I would express that I am sad about/down because of the blog post”, *r* = .56, *p* < .001).

#### Individual Costs and Collective Benefits

We used the same 15 items as in Study 5 to assess different concerns. Items tapping into individual costs and collective benefits again formed reliable scales (*α* = .78/ = .73).

### Results

#### Identification

Participants identified moderately with feminists (*M* = 3.89, *SD* = 1.75) and identified quite highly with women (*M* = 5.59, *SD* = 1.11). The two types of identification were positively correlated, *r* = .35, *p* < .001.

#### Checks

The blog post was perceived as sexist, *M* = 35.84, *SD* = 23.21; significantly different from scale midpoint, *t*(314) = 27.41, *p* < .001, *d* = 1.54 and harming efforts towards achieving gender equality, *M* =  − 44.44, *SD* = 15.39, *t*(316) =  − 51.42, *p* < .001, *d* = 2.89. Overall, participants strongly disagreed with the blog post, *M* =  − 40.43, *SD* = 17.69, *t*(316) =  − 40.69, *p* < . 001, *d* = 2.29.^4^

Next, we tested whether the support manipulation had been successful. As expected, participants in the support condition reported that the comments by other readers opposed the blog post more strongly (*M* =  − 3.15, *SD* = 19.50), compared to the no support condition (*M* = 39.84, *SD* = 25.76), *F*(1,313) = 277.08, *p* < .001, *η*_p_^2^ = .47 (note that the skewness towards positive values is best explained by the two supporting comments that all participants read). Moreover, participants judged the comments in the support condition as more supportive of their own opinion (*M* = 0.85, *SD* = 22.86) than in the no support condition (*M* =  − 41.47, *SD* = 20.74), *F*(1,314) = 297.51, *p* < .001, *η*_p_^2^ = .49.

#### Anger About the Sexist Opinion

Participants were very angry about the blog post (*M* = 4.97, *SD* = 1.66), but, as predicted, participants reported they would not express anger to the same extent (*M* = 4.30, *SD* = 1.79). In a repeated-measures ANOVA this difference was statistically significant and, as such, provided again evidence for the anger gap, *F*(1,316) = 59.71, *p* < .001, *η*_p_^2^ = .16.

Was the size of the anger gap attenuated by feminist identification or support from other women? We added support as a between-subjects factor, and feminist identification as a continuous predictor (mean-centered) to the ANOVA model. We also included women’s identification as a control. Here, we report the results that are central to our hypotheses; all remaining results can be found in the supplementary material. We found main effects for feminist identification, *F*(1,309) = 23.38, *p* < .001, *η*_p_^2^ = .07, which was positively associated with anger, but no effect of support, *F*(1,309) = 0.36, *p* = .55, *η*_p_^2^ = .001. Critically, the anger gap remained significant, *F*(1,309) = 56.32, *p* < .001, *η*_p_^2^ = .15, while neither the Anger Gap × Support interaction, *F*(1,309) = 0.07, *p* = .80, *η*_p_^2^ < .001, nor the Anger Gap × Feminist Identification interaction, *F*(1,305) = 2.43, *p* = .12, *η*_p_^2^ = .01,^5^ reached significance. Thus, in line with Study 1 and different from our expectations, there was virtually no evidence for variation in the anger gap due to feminist identification and also support did not attenuate it.

Next, going beyond the intention to express anger, we examined how participants actually express anger in written responses. Despite having reported being very angry about the blog post, on average participants in fact expressed little anger (*M* = 1.92, *SD* = 0.68, on a scale from 1 to 4). Note also that verbally expressed anger correlated rather weakly with anger expression intentions (see Table 2).

**Table 2:** Descriptive statistics and correlations for experienced and expressed anger and sadness, individual costs, and collective benefits in Study 2

							Correlations			
		*N*	*M*	*SD*	1	2	3	4	5	6
1	Experienced Anger	317	4.97	1.66	-					
2	Experienced Sadness	317	3.32	1.54	.43**	-				
3	Anger Expression Intentions	317	4.30	1.79	.61**	.36**	-			
4	Sadness Expression Intentions	317	3.11	1.61	.31**	.63**	.46**	-		
5	Verbally Expressed Anger	283	1.94	0.68	.31**	.10	.22**	− .02	-	
6	Individual Costs	317	3.33	1.30	− .06	.13*	.05	.28**	− .21**	-
7	Collective Benefits	317	5.08	1.30	.45**	.35**	.42**	.34**	.23**	.12*

^**^p < .001; *p < .01

Did support and feminist identification affect verbally expressed anger? We tested this by running an ANOVA with support as a between-subjects factor and feminist identification as a continuous predictor (mean-centered); we added women’s identification as control. Note that the measure of verbally expressed anger cannot easily be compared to the measure of experienced anger as they not only differ in dimension (experience vs. expression), but also in format (written vs. self-report). As such, we did not consider evidence for the anger gap here, but instead controlled for experienced anger. Neither main effects nor interactions were statistically significant, *p*s > .19.

#### Costs and Benefits Concerns

Bivariate correlations (Table 2) showed that both anger expression measures were, as expected, positively associated with collective benefits. The expected negative association with individual costs was only found for verbally expressed anger. We then scrutinized unique contributions of individual costs and collective benefits concerns above and beyond anger experience in two separate multiple regressions.

For anger expression intentions, collective benefits retained a unique contribution, *B* = 0.24, *SE* = 0.07, 95% CI [0.10, 0.37], *t*(313) = 3.47, *p* = .001. Individual costs, on the other hand, did not predict anger expression intentions, *B* = 0.08, *SE* = 0.06, 95% CI [− 0.40, 0.20], *t*(313) = 1.31, *p* = .19. Unsurprisingly, experienced anger also retained a unique effect, *B* = 0.58, *SE* = 0.05, 95% CI [0.48, 0.69], *t*(313) = 11.01, *p* < .001.

Turning to verbally expressed anger, we found unique contributions of both collective benefits, *B* = 0.07, *SE* = 0.03, 95% CI [0.01, 0.14], *t*(279) = 2.18, *p* = .03, and individual costs, *B* =  − 0.11, *SE* = 0.03, 95% CI [− 0.17, − 0.05], *t*(279) =  − 3.74, *p* < .001. Here, too, experienced anger retained a unique effect, *B* = 0.10, *SE* = 0.03, 95% CI [0.05, 0.15], *t*(279) = 3.88*, p* < .001.

#### Is the Gap Emotion-specific?

As in Study 5, we tested whether the gap was specific to anger reactions or could also be observed for sadness reactions. Running a within-subjects ANOVA with the factors Gap (experience vs. expression) and Emotion (anger vs. sadness) resulted in main effects of gap, *F*(1,316) = 44.84, *p* < .001, *η*_p_^2^ = .12, and emotion, *F*(1,316) = 277.78, *p* < .001, *η*_p_^2^ = .47, and the interaction of both factors was also significant, *F*(1,316) = 21.56, *p* < .001, *η*_p_^2^ = .06. In contrast to in Study 5, subsequent simple main effects showed that the gap was larger for anger, *F*(1,317) = 59.71, *p* < .001, than for sadness, *F*(1,317) = 7.75, *p* = .01 (see Table 2 for descriptive statistics).

### Discussion

Study 2 replicated the anger gap and that expressed anger was not only predicted by experience, but also by instrumental concerns. Especially our measure of verbally expressed anger was associated with both collective benefits and individual costs. Overall, we did not find evidence that feminist identification or support by other women motivated anger expression. We found a larger gap for anger than for sadness (although the sadness gap was significant as well). This pattern is in line with our theoretical rationale, but the reverse of the pattern in Study 5 which is why we revisited the emotion-specificity of the gap in Study 3.

## Study 3

In a pre-registered study (osf.io/fvrz4), we predicted that women’s reactions show a larger gap for anger than for sadness (i.e., a replication of Study 2). Moreover, we tested whether the anger gap is group-specific by collecting data from women and men. We predicted that the anger gap is larger for women than for men and that women express less anger than men. Relatedly, women should consider individual costs more than men. Lastly, we complemented our measure of individual costs and collective benefits, with individual benefits and collective costs items, thereby fully crossing the individual/collective and costs/benefits dimensions.

### Methods

#### Participants

We recruited 258 participants through the online platform Prolific.co. The study was advertised to self-identified men and women, which was critical for the planned comparisons between groups, between the age of 18 and 30, to match the samples of the two first studies. From the initial sample, we excluded 17 participants from the analyses. Three participants did not categorize themselves as either male or female at the beginning of the study. Two participants stopped their participation on the first pages of the study, and 12 participants withdrew their agreement for their data to be used at the end of the study, leading to a final sample of 123 women and 118 men (*M*_age_ = 22.30, *SD*_age_ = 3.16). Participation was compensated with 1.90 £. The majority of participants was Polish (81; overall, participants came from 32 different countries).

A priori, we computed sample size and sensitivity analyses in G*Power (Faul et al., 2007). Based on the previous studies, we expected that the Anger Gap for women is relatively large (*f* = .44). However, as we include a new factor (gender) in this study, we decided to collect responses from enough participants to detect small effects (*f* = .10) with *α* = .05 and statistical power of 0.90. In order to detect a 2-way interaction with one within-subjects factor (assumed correlation *r* = .53, based on the lower bound 95% CI in the previous study), a sample of *N* = 250 was required. This provided sufficient power to detect small to medium effects in one-way ANOVAs to test main effects of gender (*f* = .20), correlations (*r* = .20), and regressions (with up to 7 predictors *f* = .08). As our final sample after exclusions was slightly smaller than anticipated, we re-ran sensitivity analyses for all central analyses; the results revealed virtually no deviation from our initial estimates.

#### Procedure and Design

Procedure and materials were based on those in Study 5 with some slight modifications. Participants first reported their identification with the self-identified gender (i.e., male or female) and with feminists. Next, we introduced the sexist blog post. In this context, we informed participants that we were interested in how people react to the blog post, and that they would have the opportunity to formulate a reply to the blog post later on. To increase credibility and relevance of this opportunity, we created an audience by presenting two comments below the blog post, ostensibly written by two other participants and randomly selected, and the information that the participant’s own reply could also be shown to others. The two comments were identical to those in Study 5 and expressed agreement with the blog post, thereby conveying that its stance was shared by others. Subsequently, we assessed how the blog post was perceived and how participants felt about it (i.e., emotion experience).

Next, participants gave their opinion on the comments by other participants and reported the extent to which they would express various emotions in their own reply. Participants could then write their own reply to the blog post or forgo this opportunity. As in Study 2, expressed anger in written replies was later rated by independent, trained raters. We then assessed concerns, now covering individual and collective costs and benefits.

#### Measures

Materials were identical with those used in Studies 1 and 2 with the following adjustments and additions.

#### Identification

For female participants, we used the same items as in Studies 1 and 2 to measure women identification (*α* = .88). For male participants, we used the same items with reference to men, rather than women (*α* = .81). Both female and male participants completed the feminist identification measure (*α* = .97).

#### Anger About the Sexist Opinion

We measured *experienced anger* with three items (angry, irritated, annoyed; *α* = .95). We used the same adjectives to measure *anger expression intentions* (*α* = .94).

As in Study 2, we obtained a measure of *verbally expressed anger* from written responses. In total, 190 participants provided a reply. These differed in length from three words to 292 words, with an average length of 45.76 words (*SD* = 44.31). Two female independent raters, blind to the gender of participants, judged whether participants rated anger expression in these comments (1 *not angry at all* to 4 *very angry*). The intraclass correlation coefficient indicated that absolute agreement between both raters was moderate, *r* = .85, 95% CI [0.80, 0.89].

#### Sadness About the Sexist Opinion

We used statements including three adjectives—sad, down, and unhappy—to measure *experienced sadness* (*α* = .85) and expressed sadness (*α* = .81).

#### Costs and Benefits

As our individual costs and collective benefits measures in Studies 1 and 2 consisted of different numbers of items, we slightly adjusted both measures to consist of five items each. The resulting two scales had good reliability (for individual costs, *α* = .76; for collective benefits *α* = .86).

Moreover, we complemented the measures of individual costs and collective benefits with measures of *individual benefits* (five items, e.g., “I want to show that I am progressive”, *α* = .74) and *collective costs* (five items, e.g., “I want to avoid backlash for women” *α* = .66).

We also submitted all 20 items to an exploratory factor analysis (generalized least square extraction and oblimin rotation). Inspection of the scree-plot suggested a two-factor, rather than four-factor solution. As it was our goal to fully cross individual vs. collective and costs vs. benefits concerns and the planned facets showed acceptable to good reliability, we nonetheless retained the four-factor structure. Information on the factor analysis and an exploration of the two factors are provided in the supplementary material.

### Results

#### Checks

Overall, participants perceived the blog post as sexist, *M* = 31.39, *SD* = 30.27; significantly different from scale midpoint, *t*(240) = 16.10, *p* < .001, *d* = 1.04 and harming efforts towards achieving gender equality, *M* =  − 38.09, *SD* = 23.08; *t*(240) =  − 25.61, *p* < .001, *d* =  − 1.65. Overall, participants strongly disagreed with the blog post, *M* =  − 32.18, *SD* = 28.84; *t*(240) =  − 17.32, *p* < .001, *d* =  − 1.12. These perceptions differed depending on gender. Compared to men, women judged the blog post as more sexist, *M* = 40.22, *SD* = 21.02 vs. *M* = 22.18, *SD* = 35.37; *t*(239) =  − 4.84, *p* < .001, *d* =  − 0.62, as more harmful for efforts towards gender equality, *M* =  − 43.60, *SD* = 17.98 vs. *M* =  − 32.34, *SD* = 26.28; *t*(239) = 3.90, *p* < .001, *d* = 0.50, and generally disagreed more strongly with it, *M* =  − 41.75, *SD* = 16.79 vs. *M* =  − 22.20, *SD* = 34.87, *t*(239) = 5.58, *p* < .001, *d* = 0.72.

Moreover, as expected, the comments ostensibly written by other participants were understood as being in line with the blog post, *M* = 39.36, *SD* = 26.32; *t*(240) = 23.17, *p* < .001, *d* = 1.50 and opposing the participant’s own opinion, *M* =  − 34.36, *SD* = 28.60; *t*(240) =  − 18.65, *p* < .001, *d* =  − 1.20. Here, women and men did not differ in their perception of the comments as being in line with the blog post, *t*(238) =  − 1.40, *p* = .16, *d* =  − 0.18, but the comments were less in line with women’s own opinion (*M* =  − 42.48, *SD* = 19.31) than with men’s opinion (*M* =  − 25.90, *SD* = 33.88), *t*(239) = 4.69, *p* < .001,* d* = 0.61.

#### Anger About the Sexist Opinion

**Fig. 1:**
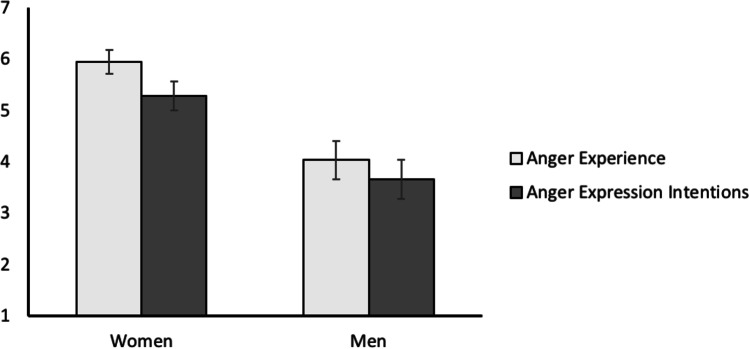
Anger gap for women and men in Study 3. Error bars indicate 95% CI

As in Studies 1 and 2, we first tested the anger gap. We ran a mixed ANOVA with the repeated self-report measure anger (experience vs. expression intentions) and gender as a between-subjects factor (men vs. women). As expected, overall, anger expression was lower than anger experience, *F*(1,239) = 52.23, *p* < .001, *η*_p_^2^ = .18, and further, gender had a main effect on overall anger (both experienced and expressed), *F*(1,239) = 67.11, *p* < .001, *η*_p_^2^ = .22. Critically, the anger gap was qualified by gender, *F*(1,239) = 4.06, *p* = .045, *η*_p_^2^ = .02. Simple main effects, as expected, revealed that the gap was larger for women, *F*(1,122) = 48.89, *p* < .001, than for men, *F*(1,117) = 11.96, *p* < .001 (see Fig. 1).^6^

Next, moving beyond expression intentions, we tested whether women and men differed in the extent to which they verbally expressed anger in their written responses. An inspection of the means indicated that women (*M* = 2.13, *SD* = 0.83) expressed more anger in their written replies to the blog post than men did (*M* = 1.70, *SD* = 0.85). However, this difference was due mostly to the fact that women *experienced* more anger to begin with—when controlling for anger experience, the gender difference in verbal expression of anger was non-significant, *F*(1,187) = 0.07, *p* = .80, *η*_p_^2^ < .001, meaning that gender did not affect the extent to which anger was expressed verbally.

#### Costs and Benefits Concerns

An inspection of bivariate correlations (Table 3) showed that both anger expression intentions and verbally expressed anger were positively associated with collective benefits and negatively with individual costs. Moreover, both anger expression measures correlated positively with the new individual benefits measure but not with the collective costs measure. We then ran multiple linear regressions to test which concerns uniquely predicted anger expression.

**Table 3:** Descriptive statistics and correlations for experienced and expressed anger and sadness as well as costs and benefits concerns in Study 3

					Correlations							
		*N*	*M*	*SD*	1	2	3	4	5	6	7	8
1	Experienced Anger	241	5.01	1.95	-							
2	Experienced Sadness	241	4.39	1.76	.80**							
3	Anger Expression Intentions	241	4.49	2.00	.84**	.70**						
4	Sadness Expression Intentions	241	3.89	1.70	.65**	.77**	.73**					
5	Verbally Expressed Anger	190	1.92	0.87	.48**	.38**	.51**	.33**				
6	Individual Costs	241	3.30	1.41	− .20**	− .05	− .14*	.09	− .24**			
7	Collective Benefits	241	5.20	1.40	.57**	.54**	.54**	.44**	.36**	.09		
8	Individual Benefits	241	4.66	1.30	.25**	.31**	.32**	.33**	.21*	.44**	.64**	
9	Collective Costs	241	3.53	1.24	.07	.18*	.12	.28**	− .02	.67**	.33**	.49**

^**^p < .001; *p < .01

For anger expression intentions, individual benefits were the only concern that emerged as a significant predictor, *B* = 0.20, *SE* = 0.08, 95% CI [0.05, 0.36], *t*(235) = 2.56, *p* = .01. Unsurprisingly, anger experience also retained a unique effect, *B* = 0.82, *SE* = 0.05, 95% CI [0.73, 0.91], *t*(235) = 18.10, *p* < .001 (individual costs, *B* =  − 0.11, *SE* = 0.07, 95% CI [− 0.25, 0.03], *t*(235) =  − 1.50, *p* = .13; collective benefits, *B* =  − 0.02, *SE* = 0.08, 95% CI [− 0.18, 0.13], *t*(235) =  − 0.27, *p* = .78; collective costs, *B* = 0.12, *SE* = 0.08, 95% CI [− 0.04, 0.28], *t*(235) = 1.45, *p* = .15).

For verbally expressed anger, both individual benefits, *B* = 0.12, *SE* = 0.06, 95% CI [0.001, 0.25], *t*(184) = 1.99, *p* = .048, and individual costs, *B* =  − 0.16, *SE* = 0.06, 95% CI [− 0.28, − 0.04], *t*(184) =  − 2.67, *p* = .01, were significant predictors. Again, anger experience also retained a unique effect, *B* = 0.15, *SE* = 0.04, 95% CI [0.08, 0.22], *t*(184) = 4.09, *p* < .001, collective benefits, *B* = 0.30, *SE* = 0.07, 95% CI [− 0.10, 0.16], *t*(184) = 0.45, *p* = .65; collective costs, *B* = 0.04, *SE* = 0.06, 95% CI [− 0.09, 0.16], *t*(184) = 0.61, *p* = .54.

Thus, these results suggest that individual concerns were better predictors of anger expression than collective concerns.

Were there any gender differences with regard to individual costs concerns? Other than expected, women did not take individual costs more into account than men when thinking about their reply to the blog post (*M* = 3.30, *SD* = 1.41), *F*(1,239) = 2.62, *p* = .11, *η*_p_^2^ = .01 (see the supplementary material for information on gender differences in other concerns).

We then explored whether this concern translates to lower anger expressions in the same way for women and men, or whether the association with anger expression might be stronger for women. This idea was not supported; please see the supplementary material for the analyses.

#### Emotion-specificity of the Gap

To contrast anger responses with sadness responses, we computed an Emotion (anger vs. sadness) × Gap (experience vs. expression) × Gender (male vs. female) mixed ANOVA, using the self-report measures. Critically, the 3-way interaction was not significant, *F*(1,239) = 0.92, *p* = .34, *η*_p_^2^ = .004, and the 2-way interaction Emotion × Gap was not significant either, *F*(1,239) = 0.92, *p* = .34, *η*_p_^2^ = .004, providing little support for the hypothesis that the gap is specific to (women’s) anger. Instead, we found significant main effects of emotion, *F*(1,239) = 85.46, *p* < .001, *η*_p_^2^ = .26, gap, *F*(1,239) = 79.57, *p* < .001, *η*_p_^2^ = .25, and gender, *F(*1,239) = 50.83, *p* < .001, *η*_p_^2^ = .18. Gender also qualified the effects of emotion, *F*(1,239) = 28.54, *p* < .001, *η*_p_^2^ = .11, and gap, *F*(1,239) = 8.48, *p* = .004, *η*_p_^2^ = .03.

Following up on the Emotion × Gender interaction, simple main effects showed that women differentiated more between emotions, with stronger anger than sadness reactions (across experience and expression; *M* = 5.62, *SD* = 1.41 vs. *M* = 4.60, *SD* = 1.45), *F*(1,122) = 129.07, *p* < .001, than men did (*M* = 3.85, *SD* = 2.07 vs. *M* = 3.58, *SD* = 1.41), *F*(1,117) = 9.26, *p* = .003.

Similarly, simple main effects for the Gap × Gender interaction revealed a larger gap between experience and expression, irrespectively of emotion, for women (*M* = 5.48, *SD* = 1.32 vs. *M* = 4.74, *SD* = 1.54), *F*(1,122) = 80.95, *p* < .001, than for men (*M* = 3.90, *SD* = 1.98 vs. *M* = 3.53, *SD* = 1.93), *F*(1,117) = 15.76, *p* < .001. Hence, women seemed to be more sensitive to the issues at play here than men, as their responses were more strongly affected by the manipulated factors than men’s responses.

### Discussion

We replicated the anger gap and demonstrated that the gap was larger for women than for men. Surprisingly, however, women and men did not differ in the extent to which they took individual costs into account. Moreover, for both groups, the gap was not only present in anger reactions but also in sadness reactions, a topic on which we elaborate in the General Discussion.

## General Discussion

In three studies, we found the predicted anger gap, that is, women intended to express *less* anger than they experienced in response to sexism. Critically, expressed anger was not simply a (reduced) reflection of experience; especially verbally expressed anger (Studies 2 and 3) was only moderately correlated with experienced anger, and instead additionally associated with instrumental concerns, most consistently with individual costs. The association between anger expression and collective benefits, on the other hand, was found less consistently and results of Study 3 suggest that benefits might matter also more at the individual, rather than the collective level.

Against our predictions, feminist identification or support by other women did not motivate anger expression. What might be reasons for this? First, although high identifiers are less likely to *endorse* gender role prescriptions, they might be particularly *aware* of them and the negative consequences of their violations. Thus, motivation to express anger might be counteracted by concerns not to reinforce negative stereotypes of the feminist identity they hold dear. Second, criticism of the sexist behavior that served as support may have been seen as sufficient punishment already. These speculations, however, need to be subject to further investigation.

Unexpectedly, we found the gap not only for anger, but also for sadness reactions. This could suggest a more general downregulation of the expression of (negative) emotions. As emotionality is part of a general stereotype of women (see Brody & Hall, 2010), to express both anger and sadness may confirm this stereotype, which could be aversive for some women (indeed, Study 3 showed a larger gap for women than for men, irrespectively of emotion). Still, given that anger expression was associated with emotion-specific instrumental concerns, in particular individual costs [which were not (negatively) associated with sadness expression], it seems just as plausible that both emotion reactions were regulated for different, emotion-specific reasons.

Our investigation of gender differences showed, as predicted, a more pronounced anger gap for women, compared to men. Yet, women did not report higher levels of individual costs than men. Why were also men relatively concerned with individual costs? Potentially, this is due to general likeability concerns—people want to be perceived positively and therefore are careful in the expression of negative emotion. Alternatively, men may have felt it was not their place to express anger about sexism and were concerned about being perceived negatively by other men and women alike (though possibly for different reasons).

Taken together, our results are complex, but can inform different strands of research. First, considering the communicative function of emotion expression (Fischer & Manstead, 2008; van Kleef, 2009), the identified anger gap should pose a problem for combatting sexism. While expressing anger could mobilize opposition to sexism, there appears to be a reluctance to do so. The fact that feminist identification did not attenuate the anger gap, which conflicts with the content of this identity (van Breen et al., 2017), particularly suggests that the gap is pervasive. Second, our findings add to the growing body of literature on instrumental emotion *expression* regulation (Greenaway & Kalokerinos, 2019) and suggest that different, competing concerns may operate simultaneously.

### Limitations and Future Research

While our research provided important insights into women’s anger about sexism, more work is needed to stake it out. Our results suggest that the gap may be less specific than expected: While they demonstrated that anger plays an important role in reactions to sexism, they also showed that other emotions—in particular sadness—should not be ignored. Future work should investigate instrumental concerns associated with sadness reactions (e.g., in reinforcing passive images of women) and concerns that may be relevant across (negative) emotion reactions (e.g., implied disapproval, criticism of men). Relatedly, both women and men associated anger expression with individual costs; future research should test potentially different reasons for these concerns and their sensitivity to different audiences (i.e., women vs. men; ingroup vs. outgroup). On a more general note, our investigation focused on instrumental concerns related to anger *expression*. To understand the experience-expression gap fully, it may be just as critical to investigate (instrumental) concerns related to anger *experience*. Zooming in on two-dimensional goals of experience and expression (see Greenaway & Kalokerinos, 2019) would shed more light on the nature of the gap.

### Conclusion

We demonstrated an anger gap in response to sexism which was larger for women than for men and found evidence that expressed anger was associated with instrumental concerns. Comparisons with sadness reactions showed that the gap is not specific to anger and suggested that there could be general as well as emotion-specific concerns at play. Neither feminist identification nor support by other women bridged the anger gap. Together, these results extend our knowledge of women’s (but also men’s) reactions to sexism and, more generally, highlight the importance of considering instrumental concerns to understand emotion expression better.

## Supplementary Information

Below is the link to the electronic supplementary material.

Supplementary file1 (DOCX 67 KB)

## Footnotes

1 Note that these beneficial effects only occurred in response to *pure* anger and not anger combined with contempt, a more hostile negative emotion.

2 We conducted a pretest in which participants (*N* = 17) rated the extent to which comments were in line with the blog post and their own opinion, promoted gender equality and traditional gender roles. Additionally, they rated the extremity, aggressiveness, and plausibility of the comments. As expected, author-criticizing comments were perceived as opposing the blog post but in line with the own opinion and as promoting gender equality and opposing traditional gender roles. See the supplementary material for results.

3 We explored whether the pattern of results changes in analyses with verbally expressed anger as the dependent variable when discarding hypothetical replies; this was not the case.

4 We also tested whether these perceptions were comparable across support conditions. Even though we assessed perceptions prior to introducing the manipulation we found some differences, in particular with regard to the perception of the blog post as sexist (support no, *M* = 38.59, *SD* = 19.02; support yes, *M* = 32.96, *SD* = 26.66), *F*(1,313) = 4.68, *p* = .03, *η*_p_^2^ = .02, and with regard to agreement (support no, *M* =  − 42.58, *SD* = 12.90, support yes, *M* =  − 38.21, *SD* = 21.37), *F*(1,315) = 4.90, *p* = .03, *η*_p_^2^ = .02. Controlling for these perceptions in our main analyses left the pattern of results largely unaffected, with one exception. Here, we report results without these additional covariates and flag where this was not the case (see the supplementary material for all analyses controlled for perceptions).

5 Without controlling for situation perceptions (see 17; Footnote 4), the Anger Gap × Feminist Identification interaction was significant, (1,309) = 6.17, *p* = .01, *η*_p_^2^ = .02. However, once controlling for perceptions, this effect became non-significant, as reported here.

6 We explored the role of feminist identification by re-running the mixed ANOVA and including identification as continuous predictor. As in Studies 1 and 2, feminist identification did not attenuate the anger gap. These results are provided in the supplementary material.

## Additional Information

### Data availability

Data and material have been made available on OSF https://osf.io/fvrz4/?view_only=44b3ef80a61546fcbcaf85e90b27d12f.
