# Supplementary material for: The Rocky Road from Experience to Expression of Emotions—Women’s Anger About Sexism
Source: Affect Sci. 2021 Nov 24;2(4):414–26. doi: 10.1007/s42761-021-00081-7 (PMC9383010; doi:10.1007/s42761-021-00081-7)
Supplement: Supplementary file 1 — Supplementary file1 (DOCX 67 KB) [file 42761_2021_81_MOESM1_ESM.docx]

**Online Supplement for the manuscript**

**“The Rocky Road from Experience to Expression of Emotions - Women’s Anger about Sexism”**

Submitted to Affective Science

Julia Sasse, Jolien van Breen, Russell Spears, & Ernestine Gordijn

Corresponding author: Julia Sasse, Max-Planck-Institute for Research on Collective Goods, Bonn; sasse@coll.mpg.de

All study materials, including a log file with all measures used for exploratory purposes and not reported in the manuscript, can be found on OSF^[[1]](#footnote-1)^. There, we also provide a summary of an additional study which is part of the same research agenda but not included in this manuscript. The reason for this is a more complex design, additionally exploring effects of support for the author (as an additional between-subjects factor) which is beyond the scope of this manuscript. Moreover, we suspect that the in-group support manipulation in Study 2 and in the additional study, though identical, may have functioned differently as the onset of the #metoo movement fell in between data collections (with the additional study being conducted first). Together, this led us to decide to exclude this study from the manuscript but report its central findings for matters of transparency.

**Study 1**

***Anger about the Sexist Opinion - Additional Results***

Here, we report the results for women’s identification which we included as a control variable in our analysis. Women’s identification showed neither a main effect on anger, *F*(1,97) = 0.01, *p* = .922, η_p_^2^ < .001, nor an interaction with the anger gap, *F*(1,97) = 0.55, *p* = .46, η_p_^2^ = .01. Also, the three-way interaction Anger Gap x Feminist Identification x Women’s Identification was not significant, *F*(1,97) = 3.10, *p* = .08, η_p_^2^ = .03.

***Additional Emotions***

In addition to anger and sadness, we measured four additional emotions, namely fury, humiliation, and amusement. We selected them because we assumed that they are also likely to occur in response witnessed sexism, while, at the same time, they should not share the conflicting properties of anger: Fury should be perceived as leading to rather destructive reactions and thus hamper collective benefits. Sadness, amusement, and humiliation on the other hand may be seen as less likely to be socially sanctioned but also less effective in stimulating change.

We tested this by inspecting the correlation between the expression of the additional emotions and individual costs and collective benefits (Table S1). In line with our assumption we did not find any significant correlations for sadness, humiliation, and amusement. Expressed fury, however, was positively correlated with collective benefits.

***Additional Concerns***

As mentioned in the manuscript, participants responded to several additional concern items. In Table S1, we report descriptive statistics for these measures and their correlations with emotion measures. Critically, only the concern that voicing one’s opinion would make them feel better positively correlated with participants’ anger expression.

**Study 2**

***Pretest***

We conducted a pretest with female participants (*N* = 17) to ensure that the comments were appropriate to manipulate criticism of the sexist blogpost voiced by other women, intended to serve as support. In total, we tested four criticizing comments. First, participants read the sexist blogpost. Then, subsequently, we presented them with the ostensible replies to the blogpost. Using single item measures, participants rated the extent to which each reply was in line with the blog post and they personally agreed with it and promoted gender equality and traditional gender roles (scales ranged from -50 to +50 with higher values indicating more agreement). Additionally, they rated the extremity, aggressiveness, and plausibility of each reply (scale from 1 to 7).

The two replies selected for the support manipulation in Study 2 (Reply 1: “This post is unacceptable! It’s 2016, do you really think women still accept such nonsense?!?; Reply 2 “Jeff, guess what, we are not here to ”serve” you!!! We deserve the same opportunities as men…“) were evaluated as intended (see Table S2 for the results of one-sample t-tests, comparing ratings to the scale midpoints). Each comment was seen as opposing the blog post but in line with the own opinion. Moreover, the content was perceived as promoting equality and not traditional gender roles. Critically, both comments were judged to be highly plausible responses by women and moderately aggressive and extreme.

***Additional Emotions***

Similar to Study 1 we assessed the experience and expression of several additional emotions (humiliation, fury, amusement, happiness; two items per emotions) Descriptive statistics and correlations (across conditions) for all emotions and motives can be found in Table S3.

Surprisingly, collective benefits correlated positively with expressed sadness, humiliation, fury, but negatively with expressed amusement. Expressed sadness and humiliation were moreover positively correlated with individual costs.

***Additional Concerns***

As in Study 1, the concern that voicing one’s opinion would make them feel better positively correlated with participants’ intended expressed anger as well as verbally expressed anger. Interestingly, both expressed anger measures were also positively correlated with the goal to avoid being seen as weak or as a traditional woman.

***Anger about the Sexist Opinion – Additional Results***

Similar to feminist identification, we found a main effect of women’s identification on anger, *F*(1,309) = 8.31, *p* = .004, η_p_^2^ = .03, with anger being higher the more participants identified with women.

The Anger Gap x Support x Feminist Identification interaction did not reach significance, *F*(1,309) = 1.28, *p* = .26, η_p_^2^ = .004. Still, the Support x Feminist Identification interaction was significant, *F*(1,309) = 6.10, *p* = .01, η_p_^2^ = .02, but as support was only introduced *after* we had measured anger experience, this interaction can only be meaningfully interpreted with anger expression intentions as dependent variable. To this end, we ran a between-subjects ANCOVA, controlling for anger experience. Here, the Support x Feminist Identification interaction was not significant, *F*(1,308) = 3.65, *p* = .06, η_p_^2^ = .01.

***Central Analyses with Checks as Covariates***

Since we found differences between support conditions with regard to the perception of the blog post as sexist (no support *M* = 38.59, SD = 19.02, yes support, *M* = 32.96, *SD* = 26.66), *F*(1,313) = 4.68, *p* = .03, η_p_^2^ = .02, and with regard to agreement (no support *M* = -42.58, *SD* = 12.90, yes support *M* = -38.21, *SD* = 21.37), *F*(1,315) = 4.90, *p* = .03, η_p_^2^ = .02, we re-ran our central analyses with these two variables as covariates.

We still found a significant difference between experienced and expressed anger, *F*(1,305) = 59.77, *p* < .001, η_p_^2^ = .16, as well as the main effects for both feminist identification, *F*(1,305) = 10.88, *p* = .001, η_p_^2^ = .03, and women’s identification, *F*(1,305) = 7.54, *p* = .01, η_p_^2^ = .02. Also the Support x Feminist Identification interaction remained significant, *F*(1,305) = 6.57, *p* = .01, η_p_^2^ = .02. The Anger Gap x Feminist Identification interaction, however, was no longer significant, *F*(1,305) = 2.43, *p* = .12, η_p_^2^ = .01.

The pattern of results of the multiple regressions, scrutinizing the unique contributions of collective benefits and individual costs to expressed anger, did not change at all.

Intended expressed anger was positively predicted by collective benefits, *B* = 0.24, *SE* = .07, 95% CI [0.10, 0.37], *t*(313) = 3.45, *p* = .001, and experienced anger, *B* = 0.58, *SE* = .06, 95% CI [0.47, 0.70], *t*(313) = 9.84, *p* < .001. Individual costs remained non-significant, *B* = 0.08, *SE* = .06, 95% CI [-0.04, 0.21], *t*(313) = 1.30, *p* = .19. Moreover, neither of the controls predicted intended expressed anger, agreement, *B* = 0.004, *SE* = .01, 95% CI [-0.01, 0.02], *t*(313) = 0.84, *p* = .40, sexist, *B* = 0.003, *SE* = .004, 95% CI [-0.01, 0.01], *t*(313) = 0.77, *p* = .44.

Verbally expressed anger was positively predicted by collective benefits, *B* = 0.08, *SE* = .03, 95% CI [0.02, 0.14], *t*(283) = 2.44, *p* = .02, experienced anger, *B* = 0.07, *SE* = .03, 95% CI [0.01, 0.12], *t*(283) = 2.47, *p* < .01, and negatively by individual costs, *B* = -0.09, *SE* = .03, 95% CI [-0.15, -0.03], *t*(313) = -3.07, *p* = .002. Moreover, neither of the controls predicted intended expressed anger, agreement, *B* = -0.004, *SE* = .003, 95% CI [-0.01, 0.002], *t*(283) = -1.36, p = .17, sexist, *B* = 0.003, *SE* = .002, 95% CI [0, 0.01], *t*(283) = 1.78, *p* = .08.

**Study 3**

***Additional Emotions***

We assessed the experience and expression of several additional emotions (humiliation, fury, amusement, and happiness with two items per emotions; disappointment with three items) Descriptive statistics and correlations (across conditions) for all emotions and motives can be found in Table S4.

***Exploratory Analyses with Feminist Identification***

We explored whether feminist identification attenuated the anger gap. Not surprisingly, female participants identified more strongly with feminists than male participants (*M* = 5.08, *SD* = 1.63 vs. *M* = 2.97, *SD* = 1.71), *t*(239) = -9.79, *p* < .001, *d* = -1.26. We then re-ran the mixed ANOVA with the repeated measure anger gap (experience vs. expression) and gender as a between-subjects factor (men vs. women) and included feminist identification as well as gender identification as continuous predictors (mean centered). In line with results from Studies 1 and 2, neither the Anger Gap x Feminist Identification interaction was significant, *F*(1,232) = 1.01, *p* = .32, η_p_^2^ = .004, nor the Anger Gap x Gender x Feminist Identification interaction, *F*(1,232) = 0.45, *p* = .50, η_p_^2^ = .002. In other words, feminist identification did not attenuate the general anger gap nor specifically the anger gap for women. On the other hand, and also in line with results from the other two studies, feminist identification had a main effect on anger, *F*(1,232) = 54.83, *p* < .001, η_p_^2^ = .19.

***Costs and Benefits***

**Factor analysis.** We ran an exploratory factor analysis with generalized least square extraction and oblimin rotation on all 20 costs and benefits items. Inspection of the scree-plot suggested that a two-factor solution is to be preferred over the planned four-factor solution. These two factors may be described as capturing global benefits and global costs (Table S5). Global benefits capture progressive collective motivations (i.e., wanting to make a difference for women) as well as individual motivations (i.e., wanting to voice one’s opinion) for addressing sexism and, based on our theorizing, should be positively associated with anger. Global costs, on the other hand reflect conservative concerns, also on the collective level (i.e., wanting to show that women are warm and caring) and individual level (i.e., avoiding to be seen as a trouble maker) and as such should be negatively associated with anger. Three items that loaded on this factor were excluded due to a lack of theoretical fit; while they share image concerns with some other items (i.e., avoiding to appear weak), the content of these concerns is distinct as they relate to very different stereotypes. One further item was excluded due to considerable cross-loading (see Tabachnik & Fidell, 2012).

**Exploration 2-factor structure.** We explored the associations between anger expression and global costs and global benefits. Global costs did not correlate with anger expression intentions (*r* = .01, *p* = .83) nor with verbally expressed anger (*r* = -.11, *p* = .14) . Global benefits, on the other hand, correlated positively with both measures (*r* = .52, p < .001/ *r* = .36, p < .001). Running a multiple linear regression with anger expression intentions as outcome, neither global costs, *B* = 0.05, *SE* = .06, 95% CI [-0.07, 0.17], *t*(237) = 0.09, *p* = .38, nor collective benefits, *B* = 0.12, *SE* = .07, 95% CI [-0.01, 0.25], *t*(237) = 1.83, *p* = .07, were unique predictors. Similarly, when using verbally expressed anger as outcome, global benefits were no longer associated with expressed anger, *B* = 0.11, *SE* = .07, 95% CI [-0.02, 0.24], *t*(186) = 1.67, *p* = .10, and the effect of global costs remained non-significant, *B* = -0.12, *SE* = .05, 95% CI [-0.16, 0.03], *t*(186) = -1.41, *p* = .16.

Similar to individual costs and collective benefits, we did not find an effect of gender on global costs, *F*(1,239) = 1.15, *p* = .29, η_p_^2^ = .01, but on global benefits, *F*(1,239) = 21.17, *p* < .001, η_p_^2^ = .08. Again, female participants were more concerned about global benefits (*M* = 5.62, *SD* = 1.20) than male participants (*M* = 4.86, *SD* = 1.34).

**Gender differences.** Similar to individual costs, there were no considerable differences regarding collective costs (*M* = 3.48, *SD* = 1.29 vs. 3.58, *SD* = 1.18), *F*(1,239) = 0.36, *p* = .55, η_p_^2^ = .001. However, female participants reported higher concerns for collective benefits than male participants (*M* = 5.61, *SD* = 1.26 vs. *M* = 4.78, *SD* = 1.43), *F*(1,239) = 23.33, *p* < .001, η_p_^2^ = .09, as well as for individual benefits (*M* = 4.87, *SD* = 1.34 vs. *M* = 4.43, *SD* = 1.22), *F*(1,239) = 6.98, *p* = .01, η_p_^2^ = .03.

**Does gender moderate the association between individual costs and verbally expressed anger?** We ran a multiple regression analysis with verbally expressed anger as outcome, individual costs, collective benefits, and anger experience as predictors, and in addition included gender and the Gender x Individual Costs interaction term. The initial pattern remained largely unchanged which means that anger experience, *B* = 0.15, *SE* = .04, 95% CI [0.07, .23], *t*(184) = 3.85 *p* < .001, and individual costs, *B* = -.09, *SE* = .04, 95% CI [-0.18, -.01], *t*(184) = -2.11, *p* = .04, remained significant predictors of anger expression and, critically, the Gender x Individual costs interaction term was not significant, *B* = 0.02, *SE* = .08, 95% CI [-0.19, .14], *t*(184) = -0.27, *p* = .79. Hence, gender did not moderate the association between individual costs and collective benefits.

| Table S1  Descriptive statistics and correlations of all emotion measures (experienced and expressed) and concerns in Study 1. | | | | | | | | | | | | | | | | | | | |
| --- | --- | --- | --- | --- | --- | --- | --- | --- | --- | --- | --- | --- | --- | --- | --- | --- | --- | --- | --- |
|  |  |  |  |  | Correlations | | | | | | | | | | | | | | |
|  |  | *N* | *M* | *SD* | 1 | 2 | 3 | 4 | 5 | 6 | 7 | 8 | 9 | 10 | 11 | 12 | 13 | 14 | 15 |
| 1 | Experienced anger | 103 | 5.29 | 1.68 |  |  |  |  |  |  |  |  |  |  |  |  |  |  |  |
| 2 | Experienced sadness | 103 | 4.12 | 1.94 | .45** |  |  |  |  |  |  |  |  |  |  |  |  |  |  |
| 3 | Experienced amusement | 103 | 2.96 | 1.82 | -.21* | -.04 |  |  |  |  |  |  |  |  |  |  |  |  |  |
| 4 | Experienced humiliation | 103 | 3.62 | 1.91 | .37** | .22* | -.18 |  |  |  |  |  |  |  |  |  |  |  |  |
| 5 | Experienced fury | 103 | 4.67 | 1.65 | .77** | .35** | -.31** | .34** |  |  |  |  |  |  |  |  |  |  |  |
| 6 | Anger expression int. | 102 | 4.96 | 1.79 | .69** | .35** | -.12 | .31** | .57** |  |  |  |  |  |  |  |  |  |  |
| 7 | Sadness expression int. | 102 | 3.36 | 1.88 | .22* | .51** | .16 | .25* | .06 | .24* |  |  |  |  |  |  |  |  |  |
| 8 | Amusement expression int. | 102 | 3.03 | 1.94 | -.22* | -.08 | .70** | -.22* | -.31** | -.16 | .02 |  |  |  |  |  |  |  |  |
| 9 | Humiliation expression int. | 102 | 2.75 | 1.81 | .20* | .19 | -.07 | .68** | .15 | .32** | .26** | -.15 |  |  |  |  |  |  |  |
| 10 | Fury expression int. | 102 | 4.27 | 2.04 | .89** | .30** | -.11 | .31** | .70** | .81** | .15 | -.14 | .29** |  |  |  |  |  |  |
| 11 | Prefer not to respond | 102 | 3.44 | 2.30 | -.25* | -.15 | .13 | -.22* | -.30** | -.29** | -.16 | .21* | -.11 | -.37** |  |  |  |  |  |
| 12 | Collective benefits | 100 | 5.45 | 1.15 | .55** | .32** | -.01 | .18 | .55** | .49** | .12 | -.02 | -.01 | .50** | -.31** |  |  |  |  |
| 13 | Individual costs | 100 | 3.80 | 1.29 | -.09 | -.06 | .12 | .07 | -.11 | -.29** | .06 | -.02 | .01 | -.19 | .11 | -.06 |  |  |  |
| 14 | Feeling better | 100 | 4.79 | 1.68 | .31** | .13 | .09 | .10 | .37** | .34** | -.07 | .17 | -.08 | .43** | -.32** | .54** | -.18 | 1 |  |
| 15 | Avoid being seen as traditional/ weak^a^ | 100 | 4.33 | 1.53 | .23* | .07 | -.07 | .27** | .25* | .12 | .11 | -.23* | .10 | .14 | -.22* | .36** | .39** | .27** |  |
| 16 | Intergroup harmony^b^ | 100 | 4.88 | 1.35 | -.08 | .09 | .32** | -.01 | -.05 | -.06 | .14 | .20 | .05 | -.01 | .01 | .22* | .47** | .02 | .26** |
| *Note*. ^a^ The correlation of the two items was *r* = .37. *p* < .001; ^b^The measure was composed out of three items. α= .76**;** ** *p* < .01. **p* < .05. | | | | | | | | | | | | | | | | | | | |

| Table S2 Descriptive statistics and results of one-sample t-tests for the evaluation of replies to the sexist blog post in the pretest. | | | | | | | | | | | | | |  |
| --- | --- | --- | --- | --- | --- | --- | --- | --- | --- | --- | --- | --- | --- | --- |
|  |  |  |  |  |  |  |  | | 95% CI for Mean Difference | | |  | |  |
|  |  | *M* | *SD* | *t* | df | *p* | | Mean Difference | | Lower | Upper | | Cohen's *d* | |
| Reply 1 | Relation to blog post^a^ | -42.29 | 24.16 | -7.22 | 16 | < .001 | | -42.29 | | -54.71 | -29.88 | | -1.75 | |
|  | Personal agreement^a^ | 40.00 | 12.69 | 12.99 | 16 | < .001 | | 40.00 | | 33.47 | 46.53 | | 3.15 | |
|  | Gender equality^a^ | 33.82 | 13.99 | 9.97 | 16 | < .001 | | 33.82 | | 26.63 | 41.02 | | 2.42 | |
|  | Traditional gender roles^a^ | -36.18 | 12.11 | -12.32 | 16 | < .001 | | -36.18 | | -42.40 | -29.95 | | -2.99 | |
|  | Plausibility^b^ | 5.94 | 1.14 | 7.00 | 16 | < .001 | | 1.94 | | 1.35 | 2.53 | | 1.70 | |
|  | Extremity^b^ | 3.29 | 1.96 | -1.48 | 16 | 0.157 | | -0.71 | | -1.71 | 0.30 | | -0.36 | |
|  | Aggressiveness^b^ | 4.12 | 1.62 | 0.30 | 16 | 0.768 | | 0.12 | | -0.71 | 0.95 | | 0.07 | |
| Reply 2 | Relation to blog post^a^ | -39.77 | 23.94 | -6.85 | 16 | < .001 | | -39.77 | | -52.07 | -27.46 | | -1.66 | |
|  | Personal agreement^a^ | 45.41 | 7.19 | 26.03 | 16 | < .001 | | 45.41 | | 41.71 | 49.11 | | 6.31 | |
|  | Gender equality^a^ | 42.35 | 9.19 | 19.01 | 16 | < .001 | | 42.35 | | 37.63 | 47.08 | | 4.61 | |
|  | Traditional gender roles^a^ | -39.12 | 10.06 | -16.04 | 16 | < .001 | | -39.12 | | -44.29 | -33.95 | | -3.89 | |
|  | Plausibility^b^ | 6.41 | 0.94 | 10.59 | 16 | < .001 | | 2.41 | | 1.93 | 2.90 | | 2.57 | |
|  | Extremity^b^ | 2.71 | 1.61 | -3.31 | 16 | 0.004 | | -1.29 | | -2.12 | -0.47 | | -0.80 | |
|  | Aggressiveness^b^ | 3.41 | 1.58 | -1.53 | 16 | 0.145 | | -0.59 | | -1.40 | 0.23 | | -0.37 | |
| *Note.* ^a^Items were rated on a scale from -50 to +50; for one-sample t-tests, the alternative hypothesis specifies that the mean is different from 0. ^b^Items were rated on a scale from 1 to 7; for one sample t-tests, the alternative hypothesis specifies that the mean is different from 4 | | | | | | | | | | | | | |  |

| Table S3  Descriptive statistics and correlations of all emotion measures (experienced and expressed) and concerns in Study 2. | | | | | | | | | | | | | | | | | | | | | | | | |
| --- | --- | --- | --- | --- | --- | --- | --- | --- | --- | --- | --- | --- | --- | --- | --- | --- | --- | --- | --- | --- | --- | --- | --- | --- |
|  |  | *N* | *M* | *SD* | 1 | 2 | 3 | 4 | 5 | 6 | 7 | 8 | 9 | 10 | 11 | 12 | 13 | 14 | | 15 | | 16 | | 17 |
| 1 | Experienced anger | 317 | 4.97 | 1.66 |  |  |  |  |  |  |  |  |  |  |  |  |  |  | |  | |  | |  |
| 2 | Experienced sadness | 317 | 3.32 | 1.54 | .43** |  |  |  |  |  |  |  |  |  |  |  |  |  | |  | |  | |  |
| 3 | Experienced amusement | 317 | 2.25 | 1.39 | -.43** | -.21** |  |  |  |  |  |  |  |  |  |  |  |  | |  | |  | |  |
| 4 | Experienced humiliation | 317 | 3.72 | 1.71 | .51** | .53** | -.27** |  |  |  |  |  |  |  |  |  |  |  | |  | |  | |  |
| 5 | Exerienced fury | 317 | 4.37 | 1.78 | .78** | .43** | -.34** | .53** |  |  |  |  |  |  |  |  |  |  | |  | |  | |  |
| 6 | Experienced happiness | 317 | 1.38 | 0.75 | -.38** | -.08 | .45** | -.15** | -.28** |  |  |  |  |  |  |  |  |  | |  | |  | |  |
| 7 | Anger expression int. | 317 | 4.30 | 1.79 | .61** | .36** | -.37** | .42** | .57** | -.25** |  |  |  |  |  |  |  |  | |  | |  | |  |
| 8 | verbally expressed anger | 317 | 1.92 | 0.68 | .32** | .11 | -.11 | .10 | .34** | -.19** | .24** |  |  |  |  |  |  |  | |  | |  | |  |
| 9 | Sadness expression int. | 317 | 3.11 | 1.61 | .31** | .63** | -.20** | .45** | .29** | -.12* | .46** | .004 |  |  |  |  |  |  | |  | |  | |  |
| 10 | Amusement expression int. | 317 | 1.99 | 1.39 | -.28** | -.11 | .62** | -.15** | -.17** | .28** | -.24** | -.02 | -.16** |  |  |  |  |  | |  | |  | |  |
| 11 | Humiliation expression int. | 317 | 2.96 | 1.67 | .33** | .48** | -.20** | .62** | .31** | -.06 | .47** | .07 | .67** | -.17** |  |  |  |  | |  | |  | |  |
| 12 | Fury expression int. | 317 | 3.57 | 1.93 | .56** | .40** | -.31** | .39** | .69** | -.19** | .78** | .21** | .46** | -.20** | .52** |  |  |  | |  | |  | |  |
| 13 | Happiness expression int. | 317 | 1.27 | 0.67 | -.15** | .06 | .23** | -.05 | -0.08 | .52** | -0.08 | -.17** | 0.07 | .31** | .03 | -.002 |  |  | |  | |  | |  |
| 14 | Individual costs | 317 | 3.33 | 1.30 | -.06 | .13* | .01 | .18** | -0.05 | .16** | 0.05 | -.19** | .28** | -.02 | .26** | -.03 | .19** |  | |  | |  | |  |
| 15 | Collective benefits | 317 | 5.08 | 1.30 | .45** | .35** | -.14* | .36** | .49** | -.18** | .42** | .24** | .34** | -.14* | .31** | .44** | -.05 | .12* | |  | |  | |  |
| 16 | Feeling better | 317 | 4.21 | 1.75 | .32** | .22** | -.09 | .30** | .35** | -.05 | .34** | .13* | .24** | -.06 | .29** | .36** | -.01 | .05 | | .59** | |  | |  |
| 17 | Avoid being seen as traditional/ weak | 317 | 3.93 | 1.74 | .19** | .25** | -.02 | .29** | .20** | .01 | .24** | .12* | .29** | -.02 | .31** | .23** | .06 | .51** | | .36** | | .27** | |  |
| 18 | Intergroup harmony | 317 | 4.79 | 1.51 | .04 | .20** | .03 | .19** | .01 | -.01 | .06 | -.06 | .30** | .03 | .24** | .001 | .06 | .43** | | .39** | | .26** | | .34** |
| *Note*. ^a^The correlation of the two items was *r* = .41. *p* < .001; ^b^The measure was composed out of three items. α= .86**;** ** *p* < .01. **p* < .05. | | | | | | | | | | | | | | | | | | |  | |  | |  |  |
|  |  |  |  |  |  |  |  |  |  |  |  |  |  |  |  |  |  |  |  | |  | |  |  |

| Table S4  Descriptive statistics and correlations of all emotion measures (experienced and expressed) and concerns in Study 3. | | | | | | | | | | | | | | | | | | | | | | |
| --- | --- | --- | --- | --- | --- | --- | --- | --- | --- | --- | --- | --- | --- | --- | --- | --- | --- | --- | --- | --- | --- | --- |
|  |  | N | M | SD | 1 | 2 | 3 | 4 | 5 | 6 | 7 | 8 | 9 | 10 | 11 | 12 | 13 | 14 | 15 | 16 | 17 | 18 |
| 1 | Experienced anger | 241 | 5.01 | 1.95 |  |  |  |  |  |  |  |  |  |  |  |  |  |  |  |  |  |  |
| 2 | Experienced sadness | 241 | 4.39 | 1.76 | .80** |  |  |  |  |  |  |  |  |  |  |  |  |  |  |  |  |  |
| 3 | Experienced amusement | 241 | 1.99 | 1.27 | -.35** | -.27** |  |  |  |  |  |  |  |  |  |  |  |  |  |  |  |  |
| 4 | Experienced humiliation | 241 | 3.76 | 1.85 | .70** | .71** | -.14* |  |  |  |  |  |  |  |  |  |  |  |  |  |  |  |
| 5 | Experienced fury | 241 | 4.46 | 2.07 | .90** | .77** | -.30** | .74** |  |  |  |  |  |  |  |  |  |  |  |  |  |  |
| 6 | Experienced happiness | 241 | 1.74 | 1.38 | -.67** | -.57** | .52** | -.39** | -.53** |  |  |  |  |  |  |  |  |  |  |  |  |  |
| 7 | Anger expression int. | 190 | 4.49 | 2.00 | .84** | .70** | -.31** | .61** | .80** | -.57** |  |  |  |  |  |  |  |  |  |  |  |  |
| 8 | Verbally expressed anger | 241 | 1.92 | 0.87 | .48** | .38** | -.24** | .30** | .45** | -.43** | .51** |  |  |  |  |  |  |  |  |  |  |  |
| 9 | Sadness expression int. | 241 | 3.80 | 1.70 | .65** | .77** | -.22** | .63** | .62** | -.48** | .73** | .33** |  |  |  |  |  |  |  |  |  |  |
| 10 | Amusement expression int. | 241 | 1.85 | 1.22 | -.29** | -.23** | .79** | -.11 | -.25** | .45** | -.24** | -.15* | -.16* |  |  |  |  |  |  |  |  |  |
| 11 | Humiliation expression int. | 241 | 3.06 | 1.79 | .57** | .63** | -.08 | .78** | .60** | -.32** | .61** | .26** | .75** | -.003 |  |  |  |  |  |  |  |  |
| 12 | Fury expression int. | 241 | 3.90 | 2.12 | .77** | .68** | -.23** | .62** | .83** | -.45** | .89** | .52** | .69** | -.17* | .65** |  |  |  |  |  |  |  |
| 13 | Happiness expression int. | 241 | 1.56 | 1.31 | -.57** | -.48** | .46** | -.32** | -.43** | .85** | -.46** | -.34** | -.38** | .47** | -.25** | -.35** |  |  |  |  |  |  |
| 14 | Collective benefits | 241 | 5.20 | 1.40 | .57** | .54** | -.14* | .48** | .59** | -.33** | .54** | .36** | .44** | -.16* | .43** | .54** | -.26** |  |  |  |  |  |
| 15 | Individual costs | 241 | 3.30 | 1.41 | -.20* | -.05 | .32** | .004 | -.18* | .29** | -.14* | -.24* | .09 | .26** | .10 | -.09 | .25** | .09 |  |  |  |  |
| 16 | Individual benefits | 241 | 4.66 | 1.30 | .25** | .31** | .07 | .30** | .29** | -.07 | .32** | .21* | .33** | .02 | .30** | .35** | -.03 | .64** | .44** |  |  |  |
| 17 | Collective costs | 241 | 3.53 | 1.24 | .05 | .18* | .21** | .19* | .10 | .10 | .12 | -.02 | .28** | .15* | .29** | .16* | .11 | .33** | .67** | .49** |  |  |
| 18 | Global costs | 241 | 3.51 | 1.25 | -.05 | .11 | .27** | .13 | -.02 | .17* | .01 | -.11 | .22** | .20* | .22** | .06 | .17* | .27** | .89** | .53** | .92** |  |
| 19 | Global benefits | 241 | 5.25 | 1.32 | .53** | .51** | -.12 | .46** | .55** | -.29** | .52** | .36** | .41** | -.14* | .41** | .53** | -.24** | .98** | .11 | .72** | .32** | .28** |
| *Note.* ** *p* < .01. **p* < .05. | | | | | | | | | | | | | | | | | | | | | | |

| Table S5  Summary of Exploratory Factor Analysis Results for Costs and Benefits Measures in Study 3. | | |
| --- | --- | --- |
|  | Global Benefits | Global Costs |
| I want to speak up against gender discrimination. | **0.89** | -0.09 |
| I want to make a difference for women. | **0.85** | 0.02 |
| I want to advance the situation for all women. | **0.77** | -0.003 |
| I want to prevent such posts about women in the future. | **0.73** | -0.03 |
| I want to voice my opinion. | **0.68** | -0.05 |
| I want to be progressive. | **0.58** | 0.30 |
| I want to shape the opinion of other readers. | **0.52** | 0.26 |
| I want to be likable. | -0.02 | **0.74** |
| I want to be agreeable. | -0.03 | **0.67** |
| I want to avoid seen as a trouble maker. | -0.11 | **0.65** |
| I want to avoid being seen as a whiner. | -0.04 | **0.63** |
| I want to show that women are warm and caring. | 0.10 | **0.58** |
| I want to avoid backlash for women. | 0.20 | **0.54** |
| I am concerned to portray women as unreasonable. | -0.03 | **0.48** |
| I want to avoid that women are disliked. | 0.23 | **0.43** |
| I worry that replying could be harmful for women. | -0.10 | **0.42** |
| I want to avoid being seen as a feminist.^a^ | **-0.38** | **0.51** |
| I want to avoid appearing weak.^b^ | 0.24 | **0.46** |
| I want to avoid being seen as traditional.^b^ | 0.31 | **0.45** |
| I want to show that I am open-minded.^b^ | 0.26 | **0.58** |
| Eigenvalues | 5.36 | 3.24 |
| % of variance explained | 26.81 | 16.18 |
| α | 0.88 | 0.82 |
| Note. One item (a) was excluded due to considerable crossloading. Three items (b) were excluded due to low theoretical fit. | | |

1. <https://osf.io/fvrz4/?view_only=44b3ef80a61546fcbcaf85e90b27d12f> [↑](#footnote-ref-1)
